# A Team Training Field Research Study: Extending a Theory of Team Development

**DOI:** 10.3389/fpsyg.2019.01480

**Published:** 2019-06-26

**Authors:** Joan H. Johnston, Henry L. Phillips, Laura M. Milham, Dawn L. Riddle, Lisa N. Townsend, Arwen H. DeCostanza, Debra J. Patton, Katherine R. Cox, Sean M. Fitzhugh

**Affiliations:** ^1^Combat Capabilities Development Command, Soldier Center, Simulation Training and Technologies Center, Orlando, FL, United States; ^2^Naval Air Warfare Center Training Systems Division, Orlando, FL, United States; ^3^CCDC, Army Research Laboratory, Human Research and Engineering Directorate, Aberdeen, MD, United States; ^4^CCDC, Human Systems Integration Division, Data and Analysis Center, Aberdeen, MD, United States

**Keywords:** team knowledge emergence, teamwork, team training, team development, field research

## Abstract

Recent advances in the science of teams have provided much insight into the important attitudes (e.g., team cohesion and efficacy), cognitions (e.g., shared team cognition), and behaviors (e.g., teamwork communications) of high performing teams and how these competencies emerge as team members interact, and appropriate measurement methods for tracking development. Numerous training interventions have been found to effectively improve these competencies, and more recently have begun addressing the problem of team dynamics. Team science researchers have increasingly called for more field studies to better understand training and team development processes in the wild and to advance the theory of team development. In addition to the difficulty of gaining access to teams that operate in isolated, confined, and extreme environments (ICE), a major practical challenge for trainers of ICE teams whose schedules are already strained is the need to prioritize the most effective strategies to optimize the time available for implementation. To address these challenges, we describe an applied research experiment that developed and evaluated an integrated team training approach to improve Tactical Combat Casualty (TC3) skills in U.S. Army squads. Findings showed that employing effective team training best practices improved learning, team cognition, emergent team processes, and performance. We recommend future research should focus on understanding the types of training strategies needed to enable teams and team leaders to develop from novices to experts. Effectively modifying training to scale it to team expertise requires more research. More laboratory and field research is needed to further develop measures of team knowledge emergence for complex task domains, and include other potential emergent factors such as team leadership and resilience. Practical implications for research include developing automated tools and technologies needed to implement training and collect team data, and employ more sensitive indicators (e.g., behavioral markers) of team attitudes, cognitions and behaviors to model the dynamics of how they naturally change over time. These tools are critical to understanding the dynamics of team development and to implement interventions that more effectively support teams as they develop over time.

## Introduction

Recent advances in the science of teams have provided much insight into the important attitudes (e.g., team cohesion and efficacy), cognitions (e.g., shared team cognition), and behaviors (e.g., teamwork communications) of high performing teams and how these competencies emerge as team members interact and communicate and appropriate measurement methods for tracking development (Marlow et al., 2018; McDaniel and Salas, 2018). Numerous training interventions have been found to effectively improve these competencies (Smith-Jentsch et al., 2008; Salas et al., 2012), and more recently have begun addressing the problem of team dynamics (Grand et al., 2016; Allen et al., 2018; Lacerenza et al., 2018). Team science researchers have increasingly called for more field studies to better understand training and team development processes in the wild and to advance the theory of team development (e.g., Kozlowski et al., 2009; Salas et al., 2017; Mathieu et al., 2018). Driskell et al. (2018) discussed the importance of conducting theory-based applied experimental research to solve real-world practical problems that expand theoretical models. They noted “what we don’t know regarding teams in extreme environments far exceeds what we do know. One reason for this is that conducting applied research on teams in extreme environments is difficult” (p. 444). In addition to the difficulty of gaining access to teams that operate in isolated, confined, and extreme environments (ICE), a major practical challenge for trainers of ICE teams whose schedules are already strained is the need to prioritize the most effective strategies to optimize the time available for implementation. In this paper we describe an applied research experiment that addressed these challenges by developing and evaluating team training for improving Tactical Combat Casualty (TC3) skills in U.S. Army squads.

Conducting casualty care in combat is the epitome of teams operating in ICE environments (Goodwin et al., 2018; Power, 2018). Becoming distracted when casualties occur on the battlefield can have catastrophic consequences, as decision making, information processing, attention, and situational awareness are impaired (Stokes and Kite, 1994). When a casualty occurs, the Army medic or Navy Corpsman may not be able to immediately respond, so instead another squad member closer to the injured may react more quickly as a first responder. But, this could result in at least two squad members being unable to respond to the tactical engagement which can put the squad’s safety at greater risk, and potentially limit its ability to achieve the tactical mission. Mission failure, as well as civilian and squad member casualties are factors that have been linked to future mental health stress management challenges in service members (Hoge et al., 2004; Grieger et al., 2006).

The command-directed casualty response system for TC3 was developed by Kotwal et al. (2011, 2013) to address the need for squads and their medics/Corpsman to effectively adapt to sudden changes in tactical priorities when squad members have to tend to casualties under fire. To reduce combat casualties, they developed procedures that specified squad interactions to be performed during the four phases of TC3: care under fire, tactical field care, casualty collection point care, and casualty evacuation. Important team interactions for casualty management include employment of effective procedures for addressing medical priorities (e.g., bleeding and suffocation), and the effective management of squad roles, precision communications, and decision making. The TC3 training program includes a Commander driven after action review (AAR) process that analyzes tactical and medical outcomes to gather and implement lessons learned for continuous systemic quality improvement. Kotwal et al. (2011) demonstrated that training resulted in a measurable reduction in Died of Wounds.

However, no TC3 training has been available for conventional forces that builds the cognitive and teamwork skills necessary to manage performance under highly stressful TC3 mission tasks. Conventional military squad training has mainly focused on battle drills for physical and mechanical aspects of combat. Live, outdoor training environments lack realistic combat casualty events, utilizing mostly training lanes and popup targets (Brimstin et al., 2015). Therefore, the Office of the Secretary of Defense sponsored the Squad Overmatch (SOvM) for TC3 training program to demonstrate that including the medic/corpsman in team training could improve the potential for saving lives on the battlefield.

A training needs analysis was conducted leveraging previous research on tactical decision making under stress (e.g., Cannon-Bowers and Salas, 1998), and critical incident interviews with Subject Matter Experts (SMEs). Based on the critical incidents of typical TC3 events, SMEs identified the task role interactions and instances of cooperation needed to effectively perform TC3 and then identified four major skill area requirements (Brimstin et al., 2015). Advanced situation awareness skills involve using cognitive and behavioral skills for pattern and threat recognition and decision making. This includes identifying and interpreting non-verbal cues in the tactical environment to determine deception; physical distances in groups to determine who is in charge; voice patterns and sweating to determine whether a person is a threat or under stress; terrain and cultural features to determine where and how people are moving and acting; and applying decision heuristics to assess any anomalies that could trigger a need to take action. Stress management skills involve using cognitive and behavioral skills to maintain tactical effectiveness under combat stress that includes application of acceptance, “what’s important now,” deliberate breathing, self-talk and buddy-talk, grounding, and personal AAR. Teamwork skills were adapted from the U.S. Navy’s Team Dimensional Training program (Townsend et al., 2016) and involve team members using information exchange, communication delivery, supporting behavior, and initiative/leadership.

Next, the SOvM TC3 training was developed that incorporated existing validated curriculum for TC3 (Kotwal et al., 2011), stress exposure training (Driskell et al., 2006), and empirically validated simulation-based training design characteristics that develop team cognition, cohesion, efficacy, team knowledge emergence (TKE), and team performance (Gabelica et al., 2016; Fernandez et al., 2017). The stress exposure training method was used as the design framework (Townsend et al., 2016) for integrating instruction and training, and to ensure team members could develop skills under stress. Classroom-based instruction provided information about the skill areas and typical stressors experienced during TC3. The TC3 task stressors were gradually increased beginning with skills practice during two simulation-based training scenarios, and then skills application during three event-based scenarios in live training at an outdoor, urban training complex comprised of buildings configured as a small village. The simulation-based training approach incorporated events in the scenarios that focused on developing effective behaviors for strategic planning, information gathering, and sharing; enabled team leaders to lead pre-briefs and AARs using a structure format focused on team competency development, engage team members in goalsetting and increase motivation (cohesion and efficacy), provide feedback and encourage team members to reflect on performance, discuss progress on goals, dealing with challenges, and identify task prioritization; and monitor team performance during exercises (Kozlowski et al., 2009; Fernandez et al., 2017). An initial evaluation of the methodology was conducted in 2015 with three U.S. Army and two U.S. Marine Corps squads at an Army post based in the Southeastern U.S. (Milham et al., 2017).

The revised ITA employed in the present study was conducted over three and one half days to ensure teams had the time needed for skill development. Compared to teams receiving 1-day of standard tactical training in an outdoor facility, ITA trained teams were expected to demonstrate: (a) more emergent team process and TC3 performance behaviors during event-based scenarios and more team self-correction behaviors during the AAR (Smith-Jentsch et al., 2008; Ceschi et al., 2014; Gabelica et al., 2016; Grand et al., 2016; Fernandez et al., 2017) (Hypothesis 1); (b) higher levels of perceived team cohesion, team efficacy, team processes, team performance, and AAR climate (Smith-Jentsch et al., 2008; DeChurch and Mesmer-Magnus, 2010; Gabelica et al., 2016; Fernandez et al., 2017) (Hypothesis 2); and higher levels of shared situation awareness (DeChurch and Mesmer-Magnus, 2010; Gabelica et al., 2016; Fernandez et al., 2017) (Hypothesis 3).

### Study Design

Random assignment of squads to condition was not possible, therefore a partial-treatment control group, with multiple post-tests, quasi-experimental design was employed (Shaddish et al., 2001). Demographic information, self-reported pre-training motivation, self-reported changes in skill levels, and tested changes in knowledge were collected to determine whether any differences between experimental and control condition participants would affect the internal validity of the study (Shaddish et al., 2001), and whether training had an effect on learning (Alvarez et al., 2004).

## Materials and Methods

### Participants

Participants were 72 male members of eight U.S. Army dismounted infantry squads. Each squad was augmented with a U.S. Army medic. Two of the squads in the control condition and one squad in the experimental condition had nine members, all of the other squads had 10 members. Data were collected during the squads’ pre-deployment training at an Army post in the southeastern U.S. and in accordance with the ARL Institutional Review Board approved protocol ARL 16-030 titled “Tactical Combat Casualty Care Training for Readiness and Resilience.” The eight squads that participated in the study were drawn from two different U.S. Army Companies, were qualified to perform their squad tasks, and were able to train with medics and learn TC3.

### Experimental Task

An overarching chronological narrative taking place over a fictional 3-week time period was used to develop two 30-min scenarios for the simulation-based training, and three 45-min scenarios for live training. Subject matter experts used the event-based approach to training method to link critical tasks, task stressors and learning objectives to task cue-strategy relationships in the scenarios that would deliberately elicit TC3, advanced situation awareness, stress management, and teamwork behaviors (Fowlkes et al., 1994). The SMEs designed the narrative that gradually increased problem complexity and TC3 stressors across the five scenarios. Stressors included combat casualties to civilians and participants, improvised explosive device explosions, and sniper fire. Squad tasks included: conducting a key leader engagement; encountering hostile actors that are observing unit movement; a complex ambush consisting of a car bomb detonation followed by a far ambush; an enemy actor that attempts a failed suicide bombing; and a sniper attack on civilians and participants. Casualty status was presented on a smart phone touch screen display worn by participants, role players and Medical Simulation Training Centers trauma mannequins. It indicated mechanism of injury, injury type and location including a realistic video of the specific wound (e.g., gunshot wound), signs and symptoms, responded to treatment provided and the individual’s tactical capabilities were displayed as a result of the specific injury (move, shoot, communicate). The display provided dynamic updates of casualty status over time. If wounds were correctly assessed and treated through self, buddy, combat life saver or medic care in a timely manner, the squad member or civilian stabilized and, if not, the display depicted a “Died of Wounds” condition.

### Integrated Training Approach

Classroom instruction focused on defining and developing team member’s declarative knowledge of the important cognitions and behaviors for each skill area. Existing knowledge and skills were refreshed (i.e., combat lifesaver skills) and new knowledge areas were introduced to emphasize the importance of teamwork and performance in each of the five skill areas. Instructors engaged participants with lecture, discussion, videos, and in-class simulations, and they emphasized the importance of teamwork and team performance. The TC3 and advanced situation awareness skills were taught on the first morning. Hands-on practice was conducted to familiarize squads with their Improved First Aid Kit II. Each Soldier used simulations of the combat application tourniquet, chest decompression needle, and the nasopharyngeal airway on a trauma mannequin with realistic blood. Video snippets were used to illustrate advanced situation awareness skills, and the importance of using teamwork behaviors to ensure advanced situation awareness information was communicated throughout the squad and higher command echelons to make timely and accurate decisions. Stress management, teamwork, and integrated AAR (IAAR) instruction were taught on the second morning. Appropriate behaviors and thought processes were modeled and communicated out loud by SMEs to improve trainee understanding of how both thoughts and actions influence stress reduction. Videos and live demonstrations of stress management skills showed how performance problems could develop from losing task focus because of combat stressors, and were followed by demonstrations of how performance could be enhanced by using coping skills. Informational cross-training and positional modeling were used to engage squad members on how teamwork can potentially facilitate or hinder each other in performing TC3 tasks; and demonstrated how tasks performed by teammates working different roles for casualty care could save lives. Demonstrations and practice scenarios were used to develop an understanding of what constitutes the IAAR, and how to conduct effective IAARs.

#### Pre-briefing and Integrated AAR

The Army standard AAR is a structured review, guided by Army doctrine, that is conducted after a training exercise. It is led by a trainer (usually the Company commander or Platoon Leader) who reviews scenario events in chronological order and discusses with the team differences between actual and expected tactical performance. Team members, or participants, provide responses to questions about what happened, why it happened, and agree on how to sustain strengths and improve performance. Although the reference doctrine has incorporated guidelines from team training research, and leader training emphasizes the use of effective dialog between team members, often, the AAR is done very quickly, and focuses on only what could have been done better, paying little attention to what was done well and why (Smith-Jentsch et al., 2008).

The prebrief and IAAR method developed for this study adapted the Army standard format and also incorporated the proven methods described above for improving team motivation, cognition and performance (Townsend et al., 2018). The U.S. Navy’s Team Dimensional Training method was adapted to ensure formative feedback was given, and to encourage self-monitoring, self-reflection, knowledge exchange, and team self-correction. The trainer was required to encourage all squad members to participate and engage with the team vice letting the squad leader do most of the talking. The IAAR began with gaining team member agreement on overall performance goals. The trainer encouraged soldiers to reconstruct scenario events using geographical maps and the VBS3 replay mode of squad member avatar movements throughout exercise. Discussions compared expected performance to actual performance and required individual accountability for task performance. Following tactical skills discussions, only the IAAR incorporated topic SMEs discussing their observations of TC3, ASA, TW, and resilience, with special emphasis on explicit discussion of the teamwork behaviors required for effective ASA, resilience and TC3. The topic SMEs used information they had recorded during the scenario using skill area observation and assessment job aids and encouraged squad members to reflect on and identify tactical triggers of good and poor team behaviors, discuss their consequences, and determine behavioral solutions. Then, the Platoon leader led the squad members in setting and documenting goals for improvement to reinforce the lessons learned and integrate them into the next mission’s planning.

#### Simulation-Based Training

The U.S. Army’s Virtual Battlespace 3 (VBS3) system was the simulation-based training environment that was used and it was configured for team training via networked, desktop PCs. It is an interactive “first-person” shooter virtual environment in which squad members verbally communicate over two channels with each other through embedded virtual radios. The same live training environment squads trained on during days 3 and 4 was modeled in the VBS3 to support skills development and transfer to the live environment. Each squad member was assigned a virtual avatar that they controlled throughout a scenario. A VBS3 controller/administrator performed scenario management throughout the scenarios and several role players managed voice and control of avatar characters in the scenarios. Following each scenario, the standard AAR involved just the trainer/Platoon Leader facilitating a 40 min discussion on tactical performance and then setting tactical performance goals for the next mission planning pre-brief. The IAAR tactical discussion was discussed for 20 min facilitated by the trainer/Platoon Leader, and the remaining IAAR was facilitated by each of the knowledge area SMEs highlighting learning objectives and engaging team members in discussions as described in the introduction. Then the trainer and SMEs led the squad members in setting and documenting goals for improvement in all topic areas that were then integrated into the next mission’s planning and scenario pre-brief.

Squad virtual interactions were automatically recorded by VBS3 for use during AARs and IAARs. Only video and audio recordings were made of the squads during the AARs and IAARs.

#### Live Training

For the live training scenarios, squad member rifles were fitted with non-intrusive simulated bullets (laser-based). The urban training environment was instrumented with simulation technologies that were triggered based on pre-determined scenario events. Non-pyrotechnical devices were used that simulated explosions for improvised explosive devices, gunshots, suicide bombs, and booby traps. Fake blood devices were employed in exploding suicide vests, improvised explosive device blast effects, and gunshot wounds with active bleeding. Role players, trauma mannequins, and squad members had simulated injuries requiring the First Aid Kit II, combat application tourniquet, chest decompression needle, the nasopharyngeal airway, occlusive dressings, and TC3 cards for reporting casualty status. Squad members interacted with various avatar simulations that required observing behaviors and cues exhibited during interactions to develop a baseline of advanced situational awareness, enable identification of tactical threats, and accomplish mission objectives. During the M1 training scenario, brief coaching pauses were conducted by an observer/controller to provide formative performance feedback to the squad members in real time. The AARs and IAARs were conducted using the same approach as described above, using recorded auditory and video snippets of the squad members moving and communicating through the urban complex performing mission tasks.

### Procedure

Four experimental condition squads (two from each Company) participated in three and one half days of the ITA and four control condition squads participated in 1 day of live training on scenarios M2 and M3. The first 2 days of the ITA involved classroom instruction in the morning and simulation based team training and IAARs in the afternoon. The live training scenarios (M1, M2, and M3) were conducted on days 3 and 4 with IAARs after each one. Due to schedule limitations, one experimental condition squad did not complete the last live scenario (M3). Control condition squads only participated in scenarios M2 and M3 during 1 day, and were led in the standard U.S. Army AAR by the 2nd Lieutenant trainer after each one. All squads participated in unrelated pre-deployment training when they were not participating in the study.

### Measures

#### Self-Report Surveys

##### Pre-training motivation

Prior to the start of all training, all participants rated their pre-training motivation on a scale of 0–100 on their perceived importance (1 item) of and willingness (1 item) to successfully complete the training (Fatkin and Hudgens, 1994).

##### Self-reported skills

Prior to the start and then after the end of all training, all participants completed a 30-item self-report survey asking them to rate their current level of skill (i.e., beginner, advanced beginner, proficient, and expert) on each of the five skill areas. This survey was developed specifically for the experiment.

##### Team attitudes

Following each scenario AAR all participants completed four team attitude questionnaires with a 6-point Likert-type response format (1 = strongly disagree, 2 = agree, 3 = neither agree or disagree, 4 = agree, and 5 = strongly agree) that asked participants to rate the degree they agreed with items written as statements. A high score indicated high levels of perceived team cohesion, efficacy, processes, and performance. All the scales were developed with input from U.S. military subject matter experts in order to establish relevant face and content validity.

The 12-item team cohesion scale asked participants how their team felt about how close a unit they were during the mission just completed (e.g., at this point in time my squad feels that we are a close-knit team). This scale was adapted from a scale developed by Orvis et al. (2005), who had based their development on Craig and Kelly (1999). A coefficient alpha of 0.95 was reported by Orvis et al. (2005), and a coefficient alpha of 0.92 was reported by Orvis et al. (2006).

The eight-item team efficacy scale asked participants how confident the squad was in its ability to successfully perform and complete future missions together (e.g., at this point in time my squad is confident that we will be able to understand the tasks at hand). This scale was adapted from a collective efficacy scale developed by Karrasch (2003) who reported an inter-item reliability of 0.93.

The 14-item team action processes scale was developed to ask participants how well they thought their team coordinated and communicated during the mission just completed (e.g., during the mission my squad exchanged information with each other so that we could work together toward mission accomplishment). Scale items were based on four team action processes identified by Marks et al. (2001), however, no previous reliability estimates have been officially published.

The five-item team performance scale asked how well participants thought their team successfully performed various goals and actions during the mission just completed (e.g., during the action phase of this mission my squad completed important execution tasks in a high quality and timely fashion). No previous reliability estimates have been officially published.

##### AAR climate

Following each scenario AAR all participants completed an 8-item AAR Climate survey that had been developed for this study. It presented each item as a 7-point rating scale with word pairs anchored at each end of the scale. They circled a number on the scale that best represented the climate established in the AAR in which they had just participated (e.g., distrustful vs. trusting).

##### Team cognition

Following each AAR all participants rated their shared situation awareness on a four point Likert-type scale that had four items asking about their squad’s ability to detect and understand cues that were presented during the scenario just completed. Matthews et al. (2002) demonstrated discriminant and convergent validity for the scale in experiments with live and virtual environments, but did not report reliability estimates.

#### Topic Knowledge Tests

Prior to and after classroom instruction, experimental condition participants completed a 58-item multiple choice test of their knowledge of each of the five skill areas. Due to scheduling constraints, control condition participants completed only a post-test after their last AAR. The test was developed specifically for this experiment.

#### Team Behavior Checklists

The SMEs used the Targeted Acceptable Responses to Generated Events or Tasks (TARGETs) method to develop structured observation checklists of behavioral markers for advanced situation awareness, teamwork, and TC3 to be collected during scenarios M2 and M3, and for IAAR behaviors following each scenario (Fowlkes et al., 1994). Fowlkes et al. (1994) reported an 89% inter-observer agreement and an internal reliability estimate (split half correlation with a Spearman–Brown correction) of 0.93.

##### Team processes

The TKE measure was created based on a combination of advanced situation awareness and teamwork markers following collection of the markers during the scenarios.

###### Advanced situation awareness

During each scenario, a SME would note on the TARGET checklistwhether or not pre-determined markers were observed. Examples of advanced situation awareness behaviors were: “the squad member verbally describes characteristics of non-verbal human cues during the key leader engagement” and “the squad member verbally describes how a person’s behavior is consistent with expectations from intelligence received.” Immediately following a scenario, the SME consulted with the SME instructors to complete the checklist. Also following the experiment the SME corrected the ratings using audio and video recordings collected during the exercises.

###### Teamwork

Two SMEs used Android tablets to record whether or not teamwork TARGET behaviors were exhibited by squad members during scenario execution. Examples of teamwork behaviors were: “information is verbally communicated among squad members about their observations of the town” (Information Exchange/Passing Information) and “other squad member(s) physically provide back-up to the squad member conducting an interview with a key person.” Following the experiment, the same SMEs reviewed their ratings together using the audio and video recordings to establish 100% consensus on the teamwork behaviors.

###### Team knowledge emergence

The TKE measure was developed based on the Grand et al. (2016) definitions of retrieval, sharing, and acknowledgment. They proposed that eight core concepts and mechanisms are needed for knowledge to effectively emerge. Data Selection occurs when a team member identifies information to be learned from the task environment. Encoding is defined as a team member transforming the observed data from the environment into internalized data. Decoding is referred to as a team member transforming knowledge received from other team members into internalized knowledge. A team member performs Integration when they transform internalized data with organized relationships into internalized knowledge. Member selection involves a team member choosing to speak to other team members and Retrieval occurs when a team member identifies internalized knowledge from memory to be shared. Sharing involves a team member communicating internalized knowledge to other team members, and Acknowledgment involves generating externalized knowledge by confirming knowledge shared by another team member is internalized.

In the present study retrieval was operationalized as advanced situation awareness behavioral markers because they fit the definition of representing internalized bits of knowledge from memory that had to be shared with other team members. Sharing was operationalized as the teamwork behavioral markers for stating priorities, providing guidance, and providing situation updates because they involved communicating an organized, and coherent collection of internalized knowledge to other team members. Acknowledgment was operationalized as the teamwork behavioral markers for backup, error correction, passing information before being asked, using available internal and external sources of information, and making complete, brief, and clear reports of information because they represent an individual generating externalized knowledge by confirming knowledge shared by another team member was internalized. For example, scenario M2, event 1 had three Retrieval, two Sharing, and two Acknowledgment behaviors. Scenario event scores were created by summing the TKE behaviors and then converting the scores to a percentage of the total possible event score.

##### Tactical combat casualty care

One SME noted on the checklist during scenario execution whether or not the behaviors were exhibited by squad members. Examples of TC3 behaviors were: “squad member provides the proper injury report (MANDOWN) to squad leader,” and “squad member(s) return fire and lay suppressive fire as needed.” Immediately following a scenario, the SME consulted with TC3 instructors to confirm accuracy of the events that occurred and then completed the checklist. Then following the experiment the SME re-checked and corrected the ratings using audio and video recordings collected during the exercises. TARGET checklists were summed to produce a total score for scenarios M2 and M3 and then scores were converted to a percentage of the total possible score.

##### Team self-correction

Two SMEs used Android tablets to record whether or not AAR behaviors were exhibited by squad members. Examples of AAR behaviors were: “key scenario events were reviewed” and “the AAR was structured around the four teamwork dimensions.” Following the experiment, the same SMEs reviewed their ratings together using the audio and video recordings to establish 100% consensus. The AAR checklists were summed to produce a total score for each AAR and then scores were converted to a percentage of the total possible score.

## Results

### Design Checks

Most of the participants in the control (91%) and experimental (97%) conditions had served between one and 16 months in their current position, with both groups about equivalent in average time served in their current position (Control: *M* = 7.7 months, range = 35 months; Experimental: *M* = 6.3 months, range = 23 months). Percentage of participants reporting training related to the SOvM curriculum, familiarity with their squad members and VBS3 training were examined. None of the participants reported having had advanced situational awareness training, about a third of the participants in each condition reported having had stress management and human performance training, and just one reported having had teamwork training. About two-thirds of the participants in both conditions reported having had Combat Lifesaver (CLS) training. Compared to the control condition, more participants in the experimental condition reported having had training in First Aid and Self-Care. The majority of participants in each condition responded “if necessary, they could correctly perform” eight CLS actions. Experimental condition participants reported having more first aid and self-care training; with about 10% more of them reporting they could correctly clear an airway, use a chest decompression needle, treat a head injury, complete a casualty card, and prepare a 9-line report. The majority of participants reported some familiarity with others in their squad, with a larger percentage in the control condition (83%) reporting squad member familiarity than in the Experimental condition (72%).

No differences were found for pre-training motivation (*p* > 0.05) with both groups reporting about the same high levels of willingness to participate (Experimental: *M* = 91.39, *SD* = 12.31, *n* = 35; control: *M* = 90.14, *SD* = 16.68, *n* = 36) and moderate levels of training importance (Experimental: *M* = 67.22, *SD* = 23.55, *n* = 35; control: *M* = 72.08, *SD* = 28.14, *n* = 36).

Table 1 presents the results of a repeated measures ANOVA which indicated a main effect of condition, with experimental participants reporting significantly higher skill levels for all learning topics than the control condition participants. In addition, an interaction effect was found, with experimental condition participants reporting significantly greater gains in their knowledge of teamwork [*F*(1,68) = 19.65, *p* < 0.001, η = 0.238] and integrated AAR [*F*(1,68) = 18.46, *p* < 0.001, η = 0.214]. *Post hoc* analyses showed all participants reported they had developed significantly greater knowledge for all topic areas [TC3: *F*(1,70) = 27.70, *p* < 0.001, η = 0.284; advanced situation awareness: *F*(1,70) = 16.89, *p* < 0.001, η = 0.194; stress management: *F*(1,70) = 14.74, *p* < 0.001, η = 0.174; teamwork: *F*(1,68) = 51.74, *p* < 0.001, η = 0.432; and integrated AAR: *F*(1,68) = 37.30, *p* < 0.001, η = 0.354].

**TABLE 1:** Overall main effect of condition on self-reported skills following training.

	**Control**				**Experimental**								
	**Pre-training**		**Post-training**		**Pre-training**		**Post-training**				
			
	***M* (*n*)**	***SD***	***M* (*n*)**	***SD***	***M* (*n*)**	***SD***	***M* (*n*)**	***SD***	***F***	***df***	***η***
TC3	29.31⁢(36)	8.78	34.03⁢(35)	8.53	33.61⁢(36)	9.16	39.53⁢(35)	8.23	7.59^*^	1,70	0.098
ASA	13.94⁢(36)	4.65	15.69⁢(35)	4.16	15.97⁢(36)	3.63	18.17⁢(35)	3.55	7.58^*^	1,70	0.098
SM	22.39⁢(36)	5.69	24.28⁢(35)	4.94	24.78⁢(36)	5.27	27.69⁢(35)	5.21	7.28^*^	1,70	0.094
TW	7.22⁢(36)	3.51	8.47⁢(35)	3.68	8.85⁢(34)	4.57	14.12⁢(33)	3.38	21.19^∗∗^	1,68	0.238
AAR	8.75⁢(36)	3.89	10.25⁢(35)	3.38	10.71⁢(34)	3.94	14.62⁢(33)	3.09	18.46^∗∗^	1,68	0.214

TC3, Tactical Combat Casualty Care; ASA, Advanced Situation Awareness; SM, Stress Management; TW, Teamwork; AAR, After Action Review. ^*^p < 0.01, ^∗∗^*p* < 0.001.

Table 2 presents changes in experimental condition pre- and post-training knowledge test scores, and a comparison of experimental and control condition post-training knowledge test scores. A dependent samples *t*-test indicated that compared to their pre-test scores, experimental condition participants had small knowledge gains in all the topics except TC3. An independent samples *t*-test indicated that compared to the control condition, experimental condition participants had significantly greater post-training knowledge of advanced situation awareness and stress management.

**TABLE 2:** Changes in experimental condition pre- and post-training knowledge test scores, and comparison of experimental and control condition post-training knowledge test scores.

	**Experimental (*n* = 36)**					**Control (*n* = 36)**			
	**Pre-training**		**Post-training**			**Post-training**			
				
	***M***	***SD***	***M***	***SD***	***t*(35)**	***M***	***SD***	***t***	***df***
*TC3*	10.78	1.46	11.25	2.94	ns	10.36	1.52	ns	70
*ASA*	4.58	2.08	7.33	2.41	–5.75^∗∗^	5.92	1.83	$-2.84^{*,**}$	$65.18^{1}$
S⁢M	10.53	2.79	12.14	3.04	–3.57^∗∗^	10.72	1.86	$-2.38^{*}$	70
T⁢W	7.83	2.48	8.53	2.50	$-2.15^{*}$	7.67	1.88	ns	70
A⁢A⁢R	2.58	1.13	3.00	0.89	$-2.21^{*}$	2.97	0.10	ns	70

TC3, Tactical Combat Casualty Care; ASA, Advanced Situation Awareness; SM, Stress Management; TW, Teamwork; and AAR, After Action Review. ^1^Levene’s test for equality of variance was significant (*F* = 4.15, *p* < 0.05). ^*^p < 0.05, ^∗∗^*p* < 0.01, ^∗∗∗^*p* < 0.001.

### Behaviors

Support for Hypothesis 1 was found for TKE, TC3, and team self-correction.

#### Team Knowledge Emergence

**FIGURE 1:**
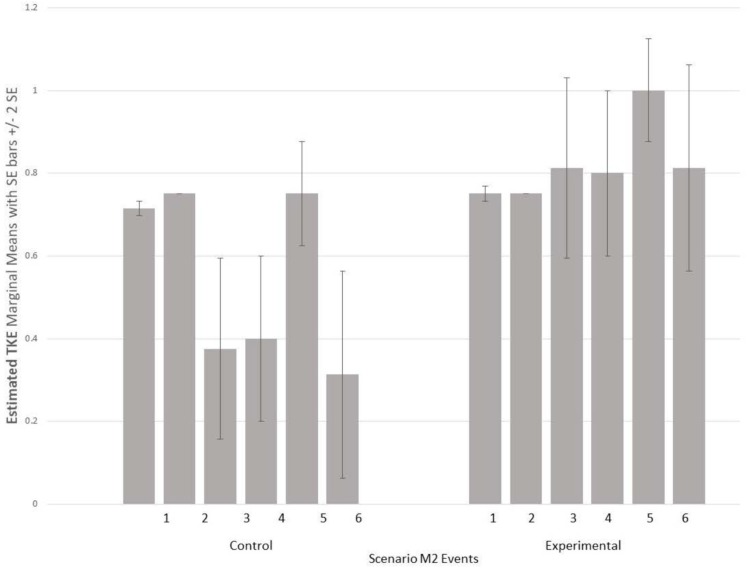
Estimated TKE marginal means and standard error for M2 scenario events.

A 2 (Condition) × 6 (Scenario Events) repeated measures ANOVA for the TKE measure indicated no interaction effect was found (*p* > 0.05), however, partial support for Hypothesis 1 was found with a main effect for condition [*F*(1,6) = 15.363, *p* < 0.01] indicating experimental condition squads demonstrated more emergent team behaviors than the control condition during scenario M2. Figure 1 shows the estimated marginal means and standard error bars for TKE at each event. Experimental condition squads maintained a higher level of team processes across the events compared to the control condition processes which diminished at scenario midpoint.

**FIGURE 2:**
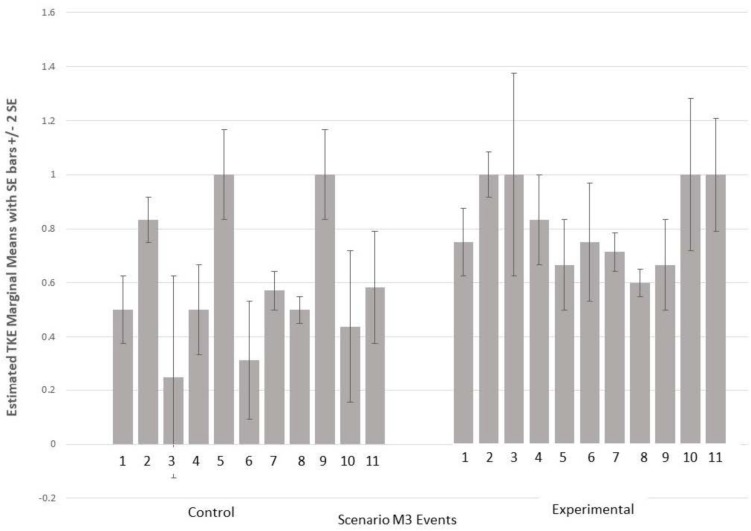
Estimated TKE marginal means and standard error for M3 scenario events.

A 2 (Condition) × 11 (Scenario Events) repeated measures ANOVA for scenario M3 indicated an interaction effect [*F*(10,50) = 2.127, *p* < 0.05], with experimental condition squads demonstrating more emergent behaviors as the events progressed. Figure 2 shows the estimated marginal means and standard error bars for TKE for each event. Similar to Figure 1, experimental condition squads maintained higher levels of team processes whereas control condition processes were lower and increased and decreased several times.

#### TC3 Performance

A 2 (Condition) × 2 (Scenario) repeated measures ANOVA indicated a main effect for condition [*F*(1,5) = 11.037, *p* < 0.05, η = 0.688] with experimental squads (*n* = 3) demonstrating more TC3 behaviors (M2: *M* = 0.550, *SD* = 0.145; M3: *M* = 0.780, *SD* = 0.225) than control condition squads (*n* = 4) (M2: *M* = 0.403, *SD* = 0.071; M3: *M* = 0.375, *SD* = 0.139). Experimental condition squads performed 15% more TC3 behaviors than the control condition during M2, and 41% more than the control condition during M3.

#### Team Self Correction

A 2 (Condition) × 2 (Scenario) repeated measures ANOVA showed a main effect for condition [*F*(1,5) = 40.961, *p* < 0.01, η = 0.891] with experimental condition squads (*n* = 3) demonstrating a larger percentage of integrated AAR behaviors (M2: *M* = 0.80, *SD* = 0.132; M3: *M* = 0.883, *SD* = 0.104) than control condition squads (*n* = 4) (M2: *M* = 0.375, *SD* = 0.087; M3: *M* = 0.450, *SD* = 0.071). Experimental condition squads performed 36% more AAR behaviors than the control condition following M2, and 43% more than the control condition following M3. A within subjects effect for scenario [*F*(1,5) = 6.289, *p* = 0.05, η = 0.557] indicated both groups demonstrated a greater percentage of integrated AAR behaviors following scenario M3 compared to scenario M2.

### Attitudes and Cognitions

Table 3 presents pooled within group correlations among team attitudes and shared situation awareness following live training scenarios M2 (Time 1) and M3 (Time 2). This correlation is calculated using only within-group sums of squares in order to avoid possible variation in scores due to the objective manipulation (ITA vs. no ITA) (Pedhazur, 1982).

**TABLE 3:** Pooled within group correlations among team attitudes and shared situation awareness following live training scenarios M2 (Time 1) and M3 (Time 2), *n* = 59.

	**T**	**Measure**	**1**	**2**	**3**	**4**	**5**	**6**	**7**	**8**	**9**	**10**	**11**	**12**
1	1	After-Action Review Climate	0.87											
2	1	Shared Situation Awareness	0.38^*^	0.76										
3	1	Team Cohesion	0.46^*^	0.29^*^	0.94									
4	1	Team Efficacy	0.55^*^	0.54^*^	0.83^*^	0.95								
5	1	Team Action Processes	0.45^*^	0.56^*^	0.67^*^	0.75^*^	0.95							
6	1	Team Performance	0.57^*^	0.48^*^	0.60^*^	0.76^*^	0.65^*^	0.88						
7	2	Team Cohesion	0.42^*^	0.37^*^	0.90^*^	0.79^*^	0.58^*^	0.47^*^	0.96					
8	2	Team Efficacy	0.46^*^	0.47^*^	0.78^*^	0.80^*^	0.56^*^	0.56^*^	0.79^*^	0.96				
9	2	Team Action Processes	0.44^*^	0.50^*^	0.74^*^	0.80^*^	0.61^*^	0.64^*^	0.76^*^	0.86^*^	0.95			
10	2	Team Performance	0.40^*^	0.52^*^	0.74^*^	0.75^*^	0.53^*^	0.63^*^	0.75^*^	0.82^*^	0.87^*^	0.91		
11	2	Shared Situation Awareness	0.18	0.46^*^	0.27^*^	0.37^*^	0.26^*^	0.38^*^	0.32^*^	0.37^*^	0.46^*^	0.58^*^	0.66	
12	2	After-Action Review Climate	0.73^*^	0.30^*^	0.28^*^	0.34^*^	0.20	0.23	0.34^*^	0.29^*^	0.30^*^	0.33^*^	0.29^*^	0.89

T, Time. Cronbach’s alpha coefficients (α) for measures are listed in the diagonal cells. ^*^p < 0.05.

No support was found for Hypothesis 2. No differences were found between conditions for team cohesion, efficacy, action processes, or performance (*p*’s > 0.05). However, Table 4 shows a significant main effect of scenario for all measures, with all participants reporting high levels of team cohesion, efficacy, processes and performance that increased slightly from scenario M2 to M3. Table 3 shows high levels of internal consistency reliability estimates, and some evidence for validity is indicated by a strong relationship between the same measures at Times 1 and 2, and somewhat smaller relationships among the different measures.

**TABLE 4:** Overall main effect of scenario on changes in team attitudes.

	**M2 (*n* = 60)**		**M3 (*n* = 60)**			
	***M***	***SD***	***M***	***SD***	***F*(1,58)**	***η***
Cohesion	4.31	0.51	4.41	0.55	8.14^∗∗^	0.123
Efficacy	4.25	0.51	4.35	0.51	5.04^*^	0.080
Action processes	4.04	0.55	4.27	0.47	$14.01^{*,**}$	0.195
Performance	4.03	0.61	4.27	0.57	12.70^∗∗^	0.180

^*^p < 0.05, ^∗∗^*p* < 0.01, ^∗∗∗^*p* < 0.001.

A 2 (Condition) × 2 (Scenario) repeated measures ANOVA for AAR climate indicated no differences (*p* > 0.20), with control condition participants (*n* = 36) (M2: *M =* 46.92, *SD* = 5.28; M3: *M =* 46.89, *SD* = 5.99) and experimental condition participants (*n* = 28) (M2: *M =* 47.96, *SD* = 6.39; M3: *M =* 48.82, *SD* = 5.85) reporting moderate to very positive reactions to the AARs. Table 3 shows high internal consistency reliability estimates at Times 1 and 2. Some evidence for validity is indicated by the strong relationship between the same measures taken at Time 1 and Time 2, and moderate relationships with the team attitude measures.

Support was found for Hypothesis 3. A 2 (Condition) × 2 (Scenario) repeated measures ANOVA indicated a between subjects effect [*F*(1,61) = 7.59, *p* < 0.01, η = 0.111]. Overall, experimental condition participants (*M =* 3.46, *SE* = 0.06) reported significantly higher levels of shared situation awareness than control condition participants (*M =* 3.23, *SE* = 0.06). A main effect of scenario was also found [*F*(1,58) = 27.28, *p* < 0.001, η = 0.309] indicating all participants reported significantly higher levels of shared awareness after the second scenario [M2 (*n* = 63): *M =* 3.21, *SD* = 0.42; M3 (*n* = 63): *M =* 3.46, *SD* = 0.39]. Table 3 shows moderate levels of internal consistency reliability at Times 1 and 2, and some evidence for validity is indicated by a moderate relationship between the same measures at both times, and with the attitude measures.

## Discussion

This study replicated past research findings that employing effective team training best practices can improve attitudes, cognitions, and performance. This is reflected in the experimental condition having higher levels of shared situation awareness, and performing more team self-correction, process, and outcome behaviors. Furthermore, these findings provide support for a theory of TKE. The ITA enabled the experimental condition squads to perform more TKE behaviors that appeared to be more consistent across scenario events, and increase their TKE performance over time, which likely contributed to better TC3 performance than the control condition squads. Despite the control condition participants reporting greater familiarity with their squad members, and the same high levels of AAR climate as the experimental condition, they performed fewer TKE behaviors and appeared more inconsistent in performing them which likely resulted in poor team performance outcomes that did not change over time. These findings are similar to what Grand et al. (2016) found. Experimental condition teams achieved total team knowledge coverage earlier than the control condition team. The control condition information exchanges flattened out at about the halfway point in the training trials, whereas information exchanges in the experimental condition continued to increase.

The small changes in team cohesion, efficacy, action processes, and performance outcomes in both groups verifies findings by Gabelica et al. (2016), lending support to the theory that these team characteristics are also emergent. However, there is no definitive explanation for the similar changes in both groups. These were mostly intact and experienced squads that were highly motivated to participate, and had very positive perceptions about each other and their performance. By the end of training they all believed they had developed better skills. Increases in positive team attitudes and self-reported learning in the control condition squads is a good sign that even the live training alone was seen as an opportunity to learn more about their team members and the subject matter. The high levels of climate indicate that both the IAAR and standard AAR were seen as supportive of team development. The moderate correlations found among AAR climate and team attitudes support the notion that AAR method in both conditions contributed to improved team attitudes. Possibly using behavioral markers to collect efficacy and cohesion indicators could provide better insight into these team characteristics than just attitude measures (Sottilare et al., 2017).

### Study Limitations

Generalizing findings based on the small number of squads in each condition is cause for concern about the validity of the findings. It is possible that the same results might not be found in a different sample. However, similarities in past experience and training and pre-training motivation were good indicators that both groups were mostly equivalent on factors that would affect internal validity. Efforts to sample the right level of expertise in the participating squads ensured they were ready to engage in training for the third phase (learning teamwork skills) of the Kozlowski et al. (2009) team development model. It is also possible we may not have had the same result with less experienced teams which should be the subject of further study.

The effort to collect data from just eight intact teams over five consecutive weeks was a significant challenge for these researchers and there were many instances when we did not have complete control over study procedures (e.g., stopping live training for rain, equipment breaking, squads and role players diverging from scenario scripts). As discussed above, we strived to address the various methodological limitations of the study by ensuring the groups were equivalent on demographic characteristics, that any training they had beyond the study was not related to what they received in the study, and that the study training they had was going to be seen as valuable in their development, even if it was for only one day.

### Theoretical Implications and Future Research

Theories of team dynamics, team development, and theory of TKE all point to the need for future team training research to focus on understanding the types of training strategies needed to enable teams and team leaders to develop from novices to experts (Fiore and Georganta, 2017; Kozlowski and Chao, 2018). The training developed in this study would likely have been too complicated for new squads with few task work skills, and possibly not challenging enough for squads with more experience than our participants. Effectively adapting training based on team expertise requires more research. For example, Kozlowski et al. (2009) provide a detailed model of team development that could inform an approach to such training. They highlighted the importance of the team leader in their four-stage model of team development (i.e., team formation, task and role development, team development, and adaptive improvement). Detailed guidance is provided for developing the attitudes, cognitions, and behaviors needed for effective team performance at each stage, describing how team knowledge, skills, abilities and attitudes should change over time, and prescribing how the team leader’s role should adapt to these phases, moving from mentor to instructor, then coach, then to facilitator to enable team growth toward adaptability. The implication for this is a commitment to studying team training interventions over longer periods of time (Burke et al., 2017).

Extending the TKE from a highly controlled lab study to a field study of a very different and more chaotic team task enabled us to demonstrate its generalizability and value in understanding team processes. However, the TKE measure we used was limited as it represented just three of the eight core concepts described by Grand et al. (2016). More laboratory and field research is needed to further develop TKE measures for complex task domains. Furthermore, these findings indicate the need to study important constructs such as resilience and team leadership as emergent factors, and the impact of emergence on team processes and performance over time (Bowers et al., 2017).

### Practical Implications

In this study we demonstrated how to integrate classroom, simulation, live training, and an integrated AAR to improve the knowledge, attitudes, processes, and performance of real, intact teams that deal with ICE environments. We also demonstrated that team training best practices can be extended to incorporate additional learning topics such as advanced situation awareness, resilience, and TC3 to emphasize the importance of how team coordination supports improving these skill areas. The U.S. Army is continuing to develop an ITA that could be implemented within its core initial military training regimen. A series of train-the-trainer studies were conducted in 2017 and 2018 with a modified ITA that was implemented mostly by a Company’s own personnel. It is also exploring an enhanced resilience training component that incorporates the importance of team responses to extreme stress reactions within the squad (Patton et al., 2018).

A successful ITA, however, requires advances in data collection and team training technologies (Johnston et al., 2018). Collecting team process and outcome performance data with human labor is highly impractical during team training exercises; the time and cost for human labor is unsupportable. A large capability gap exists for automated tools and technologies needed to collect this data. Kozlowski and Chao (2018) and others (Sottilare et al., 2017; DeCostanza et al., 2018) discuss the need to supplement static, subjective surveys with assessment and analysis technologies (e.g., socio-metric badges) that employ more sensitive indicators (e.g., behavioral markers) of team attitudes, cognitions and behaviors, and model the dynamics of how they naturally change over time. Johnston et al. (2018) developed an instructional framework based on the Kozlowski et al. (2009) team development model that provides recommendations for how instructional and intelligent tutoring technologies could provide more effective training, as well as reduce instructor load for developing these skills. These tools and technologies are critical to understanding the dynamics of team development and to implement interventions that more effectively support teams as they develop over time.

## Ethics Statement

This study was carried out in accordance with the recommendations of the U.S. Army Research Laboratory Institutional Review Board with written informed consent from all subjects. All subjects gave written informed consent in accordance with the Declaration of Helsinki. The 16-030 protocol was approved by the U.S. Army Research Laboratory Institutional Review Board.

## Author Contributions

All authors contributed to the conception and design of the study, wrote the first draft of the manuscript, revised the manuscript, and provided approval for publication of the content. JJ, LM, DR, LT, DP, KC, and SF organized the database, and performed the statistical analysis.

## Conflict of Interest Statement

The views, opinions, and findings contained in this article are the authors and should not be construed as official or as reflecting the views of the Department of Defense. This paper is intended to be approved for public release and unlimited distribution.
